# The Global Neurodegeneration Proteomics Consortium: biomarker and drug target discovery for common neurodegenerative diseases and aging

**DOI:** 10.1038/s41591-025-03834-0

**Published:** 2025-07-15

**Authors:** Farhad Imam, Rowan Saloner, Jacob W. Vogel, Varsha Krish, Gamal Abdel-Azim, Muhammad Ali, Lijun An, Federica Anastasi, David Bennett, Alexa Pichet Binette, Adam L. Boxer, Martin Bringmann, Jeffrey M. Burns, Carlos Cruchaga, Jeff L. Dage, Amelia Farinas, Luigi Ferrucci, Caitlin A. Finney, Mark Frasier, Oskar Hansson, Timothy J. Hohman, Erik C. B. Johnson, Mika Kivimaki, Roxanna Korologou-Linden, Agustin Ruiz Laza, Allan I. Levey, Inga Liepelt-Scarfone, Lina Lu, Niklas Mattsson-Carlgren, Lefkos T. Middleton, Kwangsik Nho, Hamilton Se-Hwee Oh, Ronald C. Petersen, Eric M. Reiman, Oliver Robinson, Jeffrey D. Rothstein, Andrew J. Saykin, Artur Shvetcov, Chad Slawson, Bart Smets, Marc Suárez-Calvet, Betty M. Tijms, Maarten Timmers, Fernando Vieira, Natalia Vilor-Tejedor, Pieter Jelle Visser, Keenan A. Walker, Laura M. Winchester, Tony Wyss-Coray, Chengran Yang, Niranjan Bose, Simon Lovestone, The Global Neurodegeneration Proteomics Consortium (GNPC), Farhad Imam, Rowan Saloner, Jacob W. Vogel, Varsha Krish, Gamal Abdel-Azim, Muhammad Ali, Lijun An, Federica Anastasi, David Bennett, Alexa Pichet Binette, Adam L. Boxer, Martin Bringmann, Jeffrey M. Burns, Carlos Cruchaga, Jeff L. Dage, Amelia Farinas, Luigi Ferrucci, Caitlin A. Finney, Mark Frasier, Oskar Hansson, Timothy J. Hohman, Erik C. B. Johnson, Mika Kivimaki, Roxanna Korologou-Linden, Agustin Ruiz Laza, Allan I. Levey, Inga Liepelt-Scarfone, Lina Lu, Niklas Mattsson-Carlgren, Lefkos T. Middleton, Kwangsik Nho, Hamilton Se-Hwee Oh, Ronald C. Petersen, Eric M. Reiman, Oliver Robinson, Jeffrey D. Rothstein, Andrew J. Saykin, Artur Shvetcov, Chad Slawson, Bart Smets, Marc Suárez-Calvet, Betty M. Tijms, Maarten Timmers, Fernando Vieira, Natalia Vilor-Tejedor, Pieter Jelle Visser, Keenan A. Walker, Laura M. Winchester, Tony Wyss-Coray, Chengran Yang, Niranjan Bose, Simon Lovestone

**Affiliations:** 1 Gates Ventures, Seattle, WA USA; 2 Department of Neurology, University of California, San Francisco, San Francisco, CA USA; 3 Department of Clinical Sciences Malmö, SciLifeLab, Lund University, Lund, Sweden; 4 Johnson & Johnson, Spring House, PA USA; 5 Department of Psychiatry, Washington University School of Medicine, St. Louis, MO USA; 6 NeuroGenomics and Informatics Center, Washington University School of Medicine, St. Louis, MO USA; 7 Barcelonaβeta Brain Research Center (BBRC), Pasqual Maragall Foundation, Barcelona, Spain; 8 Hospital del Mar Research Institute, Barcelona, Spain; 9 Centre for Genomic Regulation (CRG), Barcelona Institute of Science and Technology (BIST), Barcelona, Spain; 10 Department of Neurological Sciences, Rush Alzheimer’s Disease Center, Chicago, IL USA; 11 Clinical Memory Research Unit, Department of Clinical Sciences Malmö, Lund University, Lund, Sweden; 12 Department of Physiology and Pharmacology, Université de Montréal, Montreal, Quebec Canada; 13 Montreal Geriatrics Institute Research Center, Montreal, Quebec Canada; 14 University of Kansas Alzheimer’s Disease Research Center, Kansas City, KS USA; 15 Department of Neurology, University of Kansas Medical Center, Kansas City, KS USA; 16 Department of Neurology, Washington University School of Medicine, St. Louis, MO USA; 17 Indiana Alzheimer’s Disease Research Center, Indianapolis, IN USA; 18 Department of Neurology, Indiana University School of Medicine, Indianapolis, IN USA; 19 Graduate Program in Neuroscience, Stanford University, Stanford, CA USA; 20 The Phil and Penny Knight Initiative for Brain Resilience, Stanford University, Stanford, CA USA; 21 Wu Tsai Neurosciences Institute, Stanford University, Stanford, CA USA; 22 Translational Gerontology Branch, National Institute on Aging, Bethesda, MD USA; 23 Neurodegeneration and Precision Medicine Research Group, Westmead Institute for Medical Research, Westmead, New South Wales Australia; 24 Faculty of Medicine and Health, University of Sydney School of Medical Sciences, Westmead, New South Wales Australia; 25 Michael J. Fox Foundation, New York, NY USA; 26 Vanderbilt Memory & Alzheimer’s Disease, Department of Neurology, Vanderbilt University Medical Center, Nashville, TN USA; 27 Vanderbilt Genetics Institute, Vanderbilt Medical Center, Nashville, TN USA; 28 Emory University School of Medicine, Atlanta, GA USA; 29 Department of Neurology, Emory University School of Medicine, Atlanta, GA USA; 30 UCL Brain Sciences, University College London, London, UK; 31 University of Helsinki, Clinicum, Helsinki, Finland; 32 Ageing & Epidemiology (AGE) Research Unit, School of Public Health, Imperial College London, London, UK; 33 Ace Alzheimer Center Barcelona, Universitat Internacional de Catalunya, Barcelona, Spain; 34 Biomedical Research Networking Centre in Neurodegenerative Diseases (CIBERNED), National Institute of Health Carlos III, Madrid, Spain; 35 Glenn Biggs Institute for Alzheimer’s & Neurodegenerative Diseases and Department of Microbiology, Immunology and Molecular Genetics, Long School of Medicine, The University of Texas Health Science Center, San Antonio, TX USA; 36 Neurodegenerative Diseases, Hertie Institute for Clinical Brain Research, Tübingen, Germany; 37 Department of Neurodegenerative Diseases, German Center of Neurodegenerative Diseases, Tübingen, Germany; 38 IB Hochschule für Gesundheit und Soziales, Standort Stuttgart, Germany; 39 Memory Clinic, Skåne University Hospital, Malmö, Sweden; 40 Department of Radiology & Imaging Sciences, Indiana University School of Medicine, Indianapolis, IN USA; 41 Mount Sinai, Icahn School of Medicine at Mount Sinai, New York, NY USA; 42 Department of Neurology, Mayo Clinic, Rochester, MN USA; 43 Banner Alzheimer’s Institute, Phoenix, AZ USA; 44 Department of Epidemiology and Biostatistics, School of Public Health, Imperial College London, London, UK; 45 Robert Packard Center for ALS Research, Johns Hopkins University, Baltimore, MD USA; 46 Department of Biochemistry and Molecular Biology, University of Kansas Medical Center, Kansas City, KS USA; 47 Johnson & Johnson, Beerse, Belgium; 48 Department of Neurology, Hospital del Mar, Barcelona, Spain; 49 Department of Neurology, Alzheimer Center Amsterdam, Amsterdam, The Netherlands; 50 Amsterdam Neuroscience, Amsterdam, The Netherlands; 51 ALS Therapy Development Institute, Cambridge, MA USA; 52 Department of Human Genetics, Radboud University Medical Center, Nijmegen, The Netherlands; 53 Alzheimer Center Limburg, School for Mental Health and Neuroscience, Maastricht University, Maastricht, The Netherlands; 54 Laboratory of Behavioral Neuroscience, National Institute on Aging, Bethesda, MD USA; 55 Department of Psychiatry, Oxford University, Oxford, UK; 56 Department of Neurology and Neurological Sciences, Stanford University School of Medicine, Standford, CA USA; 57 Johnson & Johnson, London, UK

**Keywords:** Neurodegenerative diseases, Dementia, Biomarkers

## Abstract

More than 57 million people globally suffer from neurodegenerative diseases, a figure expected to double every 20 years. Despite this growing burden, there are currently no cures, and treatment options remain limited due to disease heterogeneity, prolonged preclinical and prodromal phases, poor understanding of disease mechanisms, and diagnostic challenges. Identifying novel biomarkers is crucial for improving early detection, prognosis, staging and subtyping of these conditions. High-dimensional molecular studies in biofluids (‘omics’) offer promise for scalable biomarker discovery, but challenges in assembling large, diverse datasets hinder progress. To address this, the Global Neurodegeneration Proteomics Consortium (GNPC)—a public–private partnership—established one of the world’s largest harmonized proteomic datasets. It includes approximately 250 million unique protein measurements from multiple platforms from more than 35,000 biofluid samples (plasma, serum and cerebrospinal fluid) contributed by 23 partners, alongside associated clinical data spanning Alzheimer’s disease (AD), Parkinson’s disease (PD), frontotemporal dementia (FTD) and amyotrophic lateral sclerosis (ALS). This dataset is accessible to GNPC members via the Alzheimer’s Disease Data Initiative’s AD Workbench, a secure cloud-based environment, and will be available to the wider research community on 15 July 2025. Here we present summary analyses of the plasma proteome revealing disease-specific differential protein abundance and transdiagnostic proteomic signatures of clinical severity. Furthermore, we describe a robust plasma proteomic signature of *APOE* ε4 carriership, reproducible across AD, PD, FTD and ALS, as well as distinct patterns of organ aging across these conditions. This work demonstrates the power of international collaboration, data sharing and open science to accelerate discovery in neurodegeneration research.

## Abstract (web summary)

The largest harmonized proteomic dataset of plasma, serum and cerebrospinal fluid samples across major neurodegenerative diseases reveals both disease-specific and transdiagnostic proteomic signatures, including a robust plasma profile associated with the *APOE*ε4 genotype.

## Main

Neurodegenerative diseases, including Alzheimer’s disease (AD), Parkinson’s disease (PD), amyotrophic lateral sclerosis (ALS), frontotemporal dementia (FTD) and other related conditions, affect more than 57 million people worldwide^1^. Until recently, treatment options were limited to managing symptoms, but approvals for disease-modifying drugs for AD and genetic forms of ALS point to considerable progress^2,3^. Eventually, it may be possible to provide patients with targeted treatments, possibly in combination, that can prevent, slow, stop or reverse the progression of their disease^4–6^. However, several major obstacles have delayed the realization of this vision. First, many neurodegenerative conditions have an extended preclinical or prodromal period where diagnosis using available symptom-based assessments is either not possible or extremely difficult due to subtle manifestations that are not detectable by current clinical tools. Second, heterogeneity in the concordance between molecular pathology and clinical syndrome as well as common co-occurrence of multiple pathologies (‘co-pathology’) contribute to misdiagnosis in clinical settings. Third, additional variability exists in the rate and pattern of symptom progression within conditions, impeding efforts to accurately prognosticate disease course. These diagnostic and prognostic challenges ultimately hinder the efficacy of clinical trials and make successful treatment of patients with any approved disease-modifying therapy challenging.

Biomarkers have the potential to resolve some of these obstacles by enabling earlier diagnosis linked to pathological processes, providing methods to subtype diseases, predicting outcomes and ultimately guiding effective intervention^7,8^. They may also improve clinical trial design through precision recruitment and serve as pharmacodynamic or surrogate endpoints in experimental medicine. Recognizing this potential, the field has seen rapid advances in imaging and fluid biomarker research, leading to their growing incorporation into clinical trials and regulatory frameworks. Fluid biomarkers, in particular, offer a real-time window into brain pathology and may help bridge the longstanding disconnect between neuropathology and clinical symptoms in living patients. Reflecting this progress, clinical guidelines have begun to integrate fluid biomarkers into routine diagnostic workflows^9^. To date, fluid biomarker development has been most successful for AD, where markers of amyloid and tau pathology are now widely used. However, there remains an urgent need for reliable biomarkers of other neurodegenerative pathologies, including α-synuclein, TDP-43 and non-AD tauopathies. In addition, biomarkers that reflect non-specific but disease-relevant biological processes, such as neuroinflammation, metabolic dysregulation and vascular dysfunction, are essential to fully characterize the pathophysiological and molecular landscape of neurodegeneration.

The accelerating development of high-throughput molecular profiling technologies, combined with increasingly powerful computational tools applied to large, deeply phenotyped cohorts, is transforming the landscape of biomarker discovery^10^. Although multi-omics approaches, such as integration of genomics, transcriptomics and metabolomics, contribute to rich data-driven biomarker discovery, proteomics is uniquely positioned to impact both diagnosis and treatment of neurodegenerative disease. This is due to several key factors: (1) many clinically established biomarkers are protein based; (2) high-dimensional proteomic platforms such as SomaScan, Olink and mass spectrometry now offer sufficient depth to capture a sizable portion of the circulating proteome; and (3) protein-level changes often capture biological processes proximal to neurodegeneration, providing functional insights that are directly relevant to disease pathogenesis. Proteomic profiles derived from peripheral biofluids such as plasma and cerebrospinal fluid (CSF) not only hold promise for identifying biomarkers of disease presence and progression but also offer new avenues for therapeutic target discovery.

Robust high-dimensionality omics research in heterogeneous clinical groups necessitates the use of large datasets due to poor reproducibility of findings from single-site or smaller cohorts^11^, but the siloing of data among a fragmented research community has been a barrier to such biomarker discovery^12^. Although many research institutions and initiatives have embarked on a variety of open data efforts, there is no standard model for providing researchers with easy access to data from multiple cohorts. Moreover, the use of such multicohort sources requires data aggregation and harmonization. The genetics research community has enabled huge consortia with joint data access and collaborative analysis^13^. In the field of neurodegenerative disease, large data-sharing efforts such as the ADNI^14^, AMP-AD^15^, PPMI^16^, ALS TDI’s ARC^17^ and Answer ALS^18^ are examples of open datasets that facilitate cross-study collaborations. However, despite these highly productive examples of best practice, their disease-specific design limits identification of shared mechanisms of neurodegeneration and potential co-existing pathologies. In addition, most data have either never been shared or have been kept behind restrictive barriers to access^12^. Reasons for this include a shortage of technology solutions, a range of challenging data governance rules and privacy regimes and cultural norms and misaligned incentives among researchers, research institutions, industry and research sponsors.

The GNPC was created to systematically address these challenges to large data analysis, accelerate biomarker discovery and advance the research and development of more precise treatments for neurodegenerative disease. Our goal was to generate a large proteomics resource using available samples from established cohort studies, accompanied by a harmonized clinical dataset, and to make these data available to the scientific community in a rapid and easily accessible manner. Having reached the planned point of public data release in July 2025, the GNPC has established what it thinks is the world’s largest neurodegenerative disease-focused proteomics dataset for biomarker research, with 23 partners contributing more than 35,000 analyzed biosamples and approximately 250 million unique protein measurements with matched and harmonized clinical data. Here we summarize the GNPC version 1 (V1) dataset, together with key analysis vignettes. With the associated in-depth papers in this issue^19–21^, this serves as the beginning of the explorations into this dataset and its contribution to the field of neurodegenerative disease.

Traditional ‘on premises’ data science analyses have become more challenging as datasets have increased in size, with a corresponding increase in resources required for moving data from one location to another. Moreover, local analysis presents a challenge in ensuring data integrity, safety and confidentiality. The GNPC’s partnership with the AD Data Initiative provided the consortium with virtual access to the cloud-resident harmonized dataset with analysis workspaces via the AD Workbench^22^, a secure, cloud-based environment that is able to satisfy multiple different geographical data jurisdictions (for example, the General Data Protection Regulation (GDPR) and the Health Insurance Portability and Accountability Act (HIPAA))^23^.

For GNPC V1, we opted to use SOMAmer technology (provided by SomaLogic) as the primary proteomics platform as it was one of the broadest discovery platforms available. However, we also analyzed a subset of samples with Olink and mass spectrometry methods to allow for cross-platform comparison. In general, the GNPC’s approach is agnostic to platforms and is guided primarily by coverage, reproducibility and affordability. We also think that different platforms bring complementary information as they may measure different isoforms and/or posttranslational modifications of a given protein.

Developing the GNPC’s large dataset required addressing several barriers to open data and data sharing. To bring together data from several countries required navigating legal regimes with different requirements, including the GDPR (Europe), the Data Protection Act (United Kingdom) and HIPAA (United States). The GNPC’s legal team worked with institutions in each jurisdiction to address specific concerns and agree on a framework for data sharing that worked across the board, including collecting the data on servers physically located in Western Europe.

The first version of the harmonized data was made available to consortium members in June 2024. Analysis of the harmonized dataset is organized in four workstreams to allow members of the consortium to collaborate on areas of related interest: longitudinal profiling, cross-sectional profiling, proteogenomics and prediction modeling. Here we present the first set of analyses of the GNPC dataset, including the overarching summary analyses and, in accompanying papers, the work conducted in the GNPC workstreams during the first year of data availability.

## Results

### Initial findings from the GNPC harmonized dataset

The GNPC V1 harmonized dataset is focused on neurodegenerative diseases and, more specifically, on AD, PD, ALS, FTD and aging among 18,645 participants, drawn from 23 individual cohorts across a total of 31,083 unique peripheral plasma, serum and CSF samples, culminating in 35,056 unique proteomic assays and approximately 250 million individual protein measurements (Table 1 and Supplementary Table 1). Most of the proteomics characterization comes from the SOMAmer-based capture array (SomaScan version 4.1, version 4 and version 3 platforms), measuring approximately 7,000 (*n* = 26,458), 5,000 (*n* = 4,528) or 1,300 (*n* = 95) unique aptamers per biosample, respectively. Additionally, 1,975 of the plasma samples characterized on the version 4.1 SomaScan platform had tandem mass tag mass spectrometry performed. The harmonized dataset additionally includes 40 clinical features, including demographic data, vital signs data and clinical features collected with each blood or CSF draw (Supplementary Table 3). These aggregated and harmonized data demonstrated their value to the consortium immediately, as they served to rapidly confirm signals originally identified in smaller datasets across the entirety of the GNPC V1 dataset, thereby serving as an ‘instant validation’ resource^24,25^.

**Table 1:** GNPC contributing cohort details (*n* = 23)

Cohort name	Organization	Geography (country)	Disease area	Sample collection and study methodology
Amyloid-beta in CSF for PD (ABC-PD)	University of Tübingen	Germany	PD	Link 1, Link 2
ALLFTD	University of California, San Francisco	United States	FTD	Link 1, Link 2
ALS Therapy Development Institute	ALS Therapy Development Institute	United States	ALS	Link 1
Alzheimer and Families (ALFA)	Barcelonaβeta Brain Research Center	Spain	AD	Link 1, Link 2
Amsterdam Dementia Cohort (ADC)	Amsterdam University Medical Center	The Netherlands	FTD and AD	Link 1, Link 2
Answer-ALS	Answer ALS	United States	ALS	Link 1, Link 2
Baltimore Longitudinal Study of Aging (BLSA)	NIA-NIH	United States	Aging	Link 1, Link 2
Banner Health	Banner Health	United States	AD and PD	Link 1, Link 2, Link 3, Link 4
BioFINDER	Lund University	Sweden	AD and PD	Link 1, Link 2
Bio-Hermes	Global Alzheimerʼs Platform Foundation	United States	AD	Link 1
CHARIOT-PRO	Imperial College London	United Kingdom	AD	Link 1
Emory ADRC	Emory University	United States	AD	Link 1
European Medical Information Framework Multimodal Biomarker Discovery Study (EMIF-AD MBD)	Maastricht University	The Netherlands	AD	Link 1, Link 2, Link 3
Fundacio ACE	Fundacio ACE	Spain	Mixed ADRD	Link 1, Link 2
Indiana ADRC	Indiana University	United States	Mixed ADRD	Link 1
Kansas ADRC	University of Kansas	United States	AD	Link 1
Knight ADRC	Washington University in St. Louis	United States	Mixed ADRD	Link 1, Link 2
Mayo Clinic Study of Aging (MCSA)	Mayo Clinic	United States	AD	Link 1
Parkinson’s Progression Markers Initiative (PPMI)	Michael J. Fox Foundation	United States	PD	Link 1, Link 2
Religious Orders Study/Memory and Aging Project (ROSMAP)	Rush University	United States	AD	Link 1, Link 2, Link 3
Stanford ADRC	Stanford University	United States	AD	Link 1, Link 2
Tracking PD	Oxford University	United Kingdom	PD	Link 1
Whitehall II	University College London	United Kingdom	Aging, Mixed ADRD	Link 1, Link 2

All contributing cohorts to the V1 harmonized dataset include the name of the study, the contributing site or organization, the country where the study was conducted, the main disease area or focus of the study and the published sample collection protocols and study methodology. ADRC, Alzheimerʼs Disease Research Center; ADRD, Alzheimerʼs disease and related dementias.

To evaluate the structure and comparability of the blood-based proteomics data, we conducted a principal component analysis on plasma and serum samples (Supplementary Fig. 1). Serum samples clustered distinctly from plasma, reflecting a clear matrix effect. Among plasma samples, a modest offset was observed between the 5K and 7K SomaScan platforms, whereas EDTA and citrate plasma samples appeared largely similar. All three vignettes described below focused exclusively on plasma proteomic data, and, where relevant, platform-related differences between 5K and 7K assays were addressed using scaling or predictive modeling approaches.

To highlight the breadth and utility of the GNPC V1 resource, we present three illustrative vignettes that showcase how this harmonized dataset can be applied to address key questions in neurodegenerative disease and aging research: (1) disease-specific differential abundance profiling, (2) biological aging across organ systems and (3) protein markers of genetic risk as exemplified by the apolipoprotein E (*APOE)* genotype. As summarized below and described in more detail in the accompanying papers in this issue^19–21^, these vignettes reflect the analytical depth enabled by the GNPC and are intended to catalyze further exploration by the broader research community upon public data release.

#### Vignette 1: Human blood proteomic profiles are robustly associated with neurodegenerative diseases and clinical severity

**Fig. 1:**
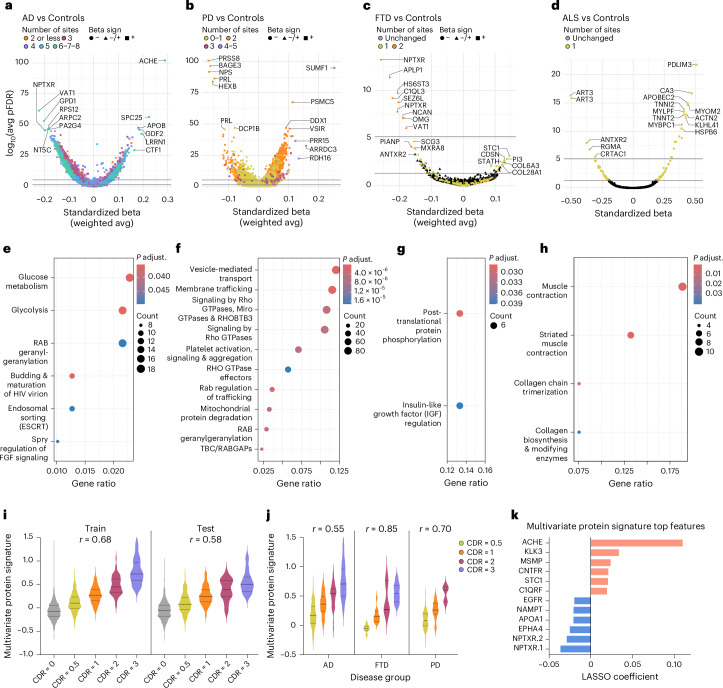
Circulating blood proteome specifies neurodegenerative disease type, mechanism and clinical severity. **a**–**d**, Meta-analytic differential abundance analysis showing changes in relative protein expression of AD (**a**), PD (**b**), FTD (**c**) and ALS (**d**) compared to Controls. Each dot represents a protein. The *x* axis shows the direction and effect size of protein changes relative to Controls, from linear regression models including age and sex as covariates; the *y* axis shows the –log_10_ FDR-adjusted *P* value. *P* values from two-sided tests and after adjustment from FDR are reported. The parallel line at the bottom of each plot shows which proteins are significant after FDR correction for multiple comparisons. The line above shows proteins further surviving Bonferroni correction. Dots are colored based on the number of cohorts where the protein was found to be independently significant after (within-cohort) FDR correction and changed in the same direction relative to Controls (that is, increased or decreased compared to Controls). **e**–**h**, Significant proteins from the differential abundance analyses were fed into Reactome enrichment analysis for AD (**e**), PD (**f**), FTD (**g**) and ALS (**h**), using unique SomaScan 7K proteins as background. Enriched Reactome pathway terms for each condition are visualized as dot plots, with dot size corresponding to the number of differentially abundant proteins assigned to a given pathway (one-sided Fisherʼs test with FDR adjustments). Full Reactome enrichment summary statistics are reported in Supplementary Table 6. HIV, human immunodeficiency virus; FGF, fibroblast growth factor; TBC/RABGAPs, Tre2–Bub2–Cdc16 (TBC) domain-containing RAB-specific GTPase-activating proteins. **i**, Violin plots displaying LASSO-derived clinical severity protein signatures across CDR global level in training and test sets. **j**, Violin plots displaying LASSO-derived clinical severity protein signatures across CDR global level (0.5 and higher) in AD, FTD and PD, using the combined training and test sets. **k**, LASSO coefficients for the top 12 protein aptamers selected in the clinical severity protein signature. avg, average; pFDR, FDR-corrected *P* value.

We examined the plasma proteome as measured using the SomaLogic 7K platform, among people with AD, PD, FTD and ALS (referred to hereafter as ‘Patients’, *n* = 3,002), and separately among people with no neurodegenerative disease diagnosis and cognitively normal test screenings (referred to hereafter as ‘Controls’, *n* = 5,879) (see Vignette 1 methods for sample selection criteria). First, we sought to identify proteins differentially abundant in the plasma of patients with different neurodegenerative diseases with cognitive effects—namely, AD, PD, FTD and ALS. Leveraging the breadth of cohorts included in the GNPC, we first performed cohort-stratified analyses to internally validate the most robust protein changes, focusing on those consistently altered across multiple study cohorts. Cohort-stratified results were subsequently combined using a meta-analysis for AD (Fig. 1a), PD (Fig. 1b), FTD (Fig. 1c) and ALS (Fig. 1d).

In AD (*n* = 1,966), 27 proteins from AD Patients robustly emerged as being significantly elevated compared to Controls across at least six of the 10 different cohorts, including ACHE, SPC25, LRRN1 and CTF1. Additionally, GDF2 and APOB also showed high meta-analytic effect sizes and were independently significant in five and four separate cohorts, respectively (Fig. 1a). In contrast, 130 proteins were consistently lower in AD plasma across at least six cohorts, including VAT1, GPD1, ARPC2 and PA2G4. Furthermore, we observed significant decreases in RPS12, NPTXR and NT5C across five cohorts. These top hits highlight both expected and underexplored targets consistently altered in AD plasma across cohorts, including those with established ties to lipid metabolism (APOB and GPD1), cholinergic signaling and/or treatment response (ACHE and VAT1) and synaptic integrity (NPTXR), as well as novel targets linked to cytoskeletal regulation (ARPC2) and RNA metabolism (PA2G4 and RPS12). Follow-up analysis (see Vignette 3) also indicated that elevation of some targets, such as SPC25, LRRN1 and CTF1, reflected underlying *APOE* ε4 genotype effects rather than AD diagnosis per se. Reactome pathway analyses (*n* = 2,640, Bonferroni-adjusted *P* < 0.05) revealed enrichment for terms related to sugar metabolism (‘glucose metabolism’ and ‘glycolysis’) and protein prenylation (‘RAB geranylgeranyltransferase’), reinforcing links to bioenergetics and vesicle trafficking (Fig. 1e).

In PD (*n* = 607), 40 proteins were significantly elevated across at least three of seven different cohorts, including SUMF1, PRR15, AARDC3 and RDH16, which were elevated in at least four cohorts (Fig. 1b). Additional proteins such as PSMC5, DDX1 and VSIR exhibited strong effect sizes and replicated in two cohorts. In contrast, 15 proteins were significantly lower in PD plasma across at least three cohorts, including CLEC3B, GPD1 and SEMA4G. Meta-analytic effect sizes were very high for PRSS8, BAGE3, NPS, PRL and HEXB, all of which decreased in PD but were less reproducible across contributing cohorts, suggesting cohort-specific factors driving depleted abundance of these targets. These candidate PD-associated proteins include targets associated with proteostatic (SUMF1, HEXB and PSMC5), immune (VSIR and CLEC3B) and axonal guidance (SEMA4G) pathways, possibly reflecting both peripheral and brain-related pathophysiology. Similar to in AD, Reactome pathway analyses (*n* = 2,251, Bonferroni-adjusted *P* < 0.05) revealed enrichment for terms related to Ras superfamily/small GTPases and vesicle trafficking (‘vesicle-mediated transport’ and ‘signaling by RHO GTPases’), highlighting a plasma proteome pathway overlap between AD and PD (Fig. 1f).

Although FTD clinical syndromes are less common than AD or PD and have greater clinical and neuropathological diversity, nine targets exhibited decreased abundance in FTD plasma (*n* = 175) across multiple cohorts after Bonferroni correction (Fig. 1c). Strongly downregulated hits included NPTXR, APLP1 and HS6ST3, which converge on processes critical for synaptic maintenance and neuronal support. Eleven proteins were significantly elevated in FTD plasma with a conventional false discovery rate (FDR) correction but did not survive Bonferroni correction. Despite limited power, Reactome pathway analyses (*n* = 71, FDR < 0.05) revealed two significantly enriched terms, ‘posttranslational protein phosphorylation’ and ‘regulation of insulin-like growth factor transport and uptake by IGFBPs’, highlighting conserved peripheral signatures of neurodegeneration even amid the clinical and pathological heterogeneity of FTD (Fig. 1g).

In ALS (*n* = 254), we analyzed plasma proteomic profiles from a single contributing cohort with Patients and Controls (Fig. 1d). After FDR correction, 44 targets exhibited significantly increased abundance in ALS, including a host of proteins related to skeletal muscle structure and function (PDLIM3, MYOM2, MYLPF and TNNI2). Thirty-eight targets exhibited significantly decreased abundance in ALS, including two aptamers targeting ART3, an ADP-ribosyltransferase enriched in skeletal muscle, as well as additional proteins linked to growth factor signaling and/or extracellular matrix composition (ANTXR2, CRTAC1 and RGMA). Reactome pathway analyses (*n* = 82, FDR < 0.05) confirmed this strong biological enrichment for skeletal muscle-related processes (‘muscle contraction’ and ‘collagen chain trimerization’) (Fig. 1h), underscoring a clear peripheral proteomic footprint of ALS consistent with its primary motor system pathology.

After identifying disease-related differential abundance patterns, we combined data across AD, PD and FTD to identify a global signature of dementia severity. Specifically, a 256-protein clinical impairment signature was derived using least absolute shrinkage and selection operator (LASSO)-based prediction of Clinical Dementia Rating (CDR) global scores, which was subsequently evaluated in a held-out test set (2,047 records; 30% of the dataset). Signature values correlated with CDR global scores (train: Pearsonʼs *r* = 0.68; test: *r* = 0.58) in a stepwise fashion, increasing at each level of clinical severity (Fig. 1i; see Supplementary Table 7 for individual-level scores). To demonstrate concordance with an orthogonal clinical outcome, we observed that higher signature values were also associated with lower cognitive test scores (standardized Montreal Cognitive Assessment/Mini-Mental State Examination (MoCA/MMSE); *r* = −0.47), consistent with expected inverse correlations between cognition and CDR. In disease-stratified analysis combined across training and test sets, the multivariate protein signature was reliably elevated with greater clinical severity in AD (*r* = 0.55), FTD (*r* = 0.85) and PD (*r* = 0.70), supporting its relevance as a transdiagnostic marker of clinical severity (Fig. 1j). Top proteins with high feature importance in the clinical impairment signature (Fig. 1k and Supplementary Table 8) again highlighted ACHE and NPTXR as well as additional targets linked to neuroplasticity (EPHA4 and CNTFR) and immune activation (MSMP and KLK3), underscoring their potential for transdiagnostic dementia staging.

#### Vignette 2: Organ age analysis reveals neurodegenerative disease-specific patterns of premature aging

**Fig. 2:**
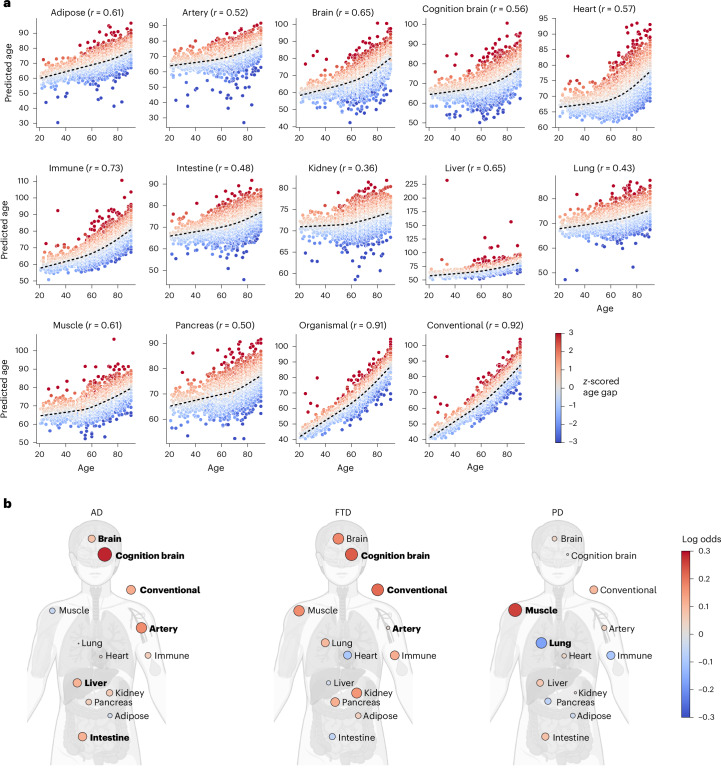
Organ age patterns characterize distinct neurodegenerative disease types. **a**, Scatterplots of chronological age versus predicted age for each organ aging clock in clinically normal individuals. Black dashed line indicates the LOWESS regression estimate of the population mean. Pearsonʼs correlation coefficient *r* is reported for each clock. **b**, Body plots showing associations of standardized organ age gaps with neurodegenerative disease based on binary logistic regression models. *P* values are from two-sided tests. Red dots indicate positive associations (higher age gap with disease); blue dots indicate negative associations (lower age gap with disease). Bold labels highlight organ ages associated with organ age gap with *P* < 0.05 after FDR correction. The body plots were created in BioRender: Oh, H. (2025): https://BioRender.com/afoqtwz.

One advantage of plasma proteomics is the ability to simultaneously query the health of distinct organ systems. We applied previously validated plasma proteomic organ aging models^26^ to assess accelerated organ-specific aging across multiple Patient and Control data in the GNPC. Predicted organ ages showed moderate to strong correlations with chronological age (*r* range, 0.36–0.92; Fig. 2a), confirming model performance in the GNPC cohort^26^. Figure 2b shows the association between organ age gaps, which capture person-specific differences between estimated organ age and actual age, and AD, FTD and PD, respectively. Elevated cognition-enriched brain age gaps, reflecting the subset of brain-specific proteins that previously enhanced model age gap prediction of cognitive impairment, were associated with higher odds of AD (odds ratio = 1.33 per 1-s.d. age gap increase (95% confidence interval: 1.25–1.41)) and FTD (odds ratio = 1.26 (95% confidence interval: 1.06–1.48)). Non-cognition-enriched brain age gap was weakly associated with AD risk (odds ratio = 1.08 (95% confidence interval: 1.02–1.14)) but not other conditions. Beyond brain, we observed contributions of artery (odds ratio = 1.18 (95% confidence interval: 1.11–1.25)), liver (odds ratio = 1.11 (95% confidence interval: 1.05–1.17)) and intestine aging to AD (odds ratio range, 1.12–1.18) as well as a unique link between muscle aging and PD (odds ratio = 1.12 (95% confidence interval: 1.05–1.19)). These findings extend previous work by demonstrating shared and distinct patterns of blood-detectable accelerated organ aging across AD, FTD and PD, underscoring connections between systemic health and neurodegenerative disease that may be related as a cause, correlate or consequence.

#### Vignette 3: Human blood proteomic signatures reflect *APOE* genetic status and uncouple systemic AD and *APOE* effects

**Fig. 3:**
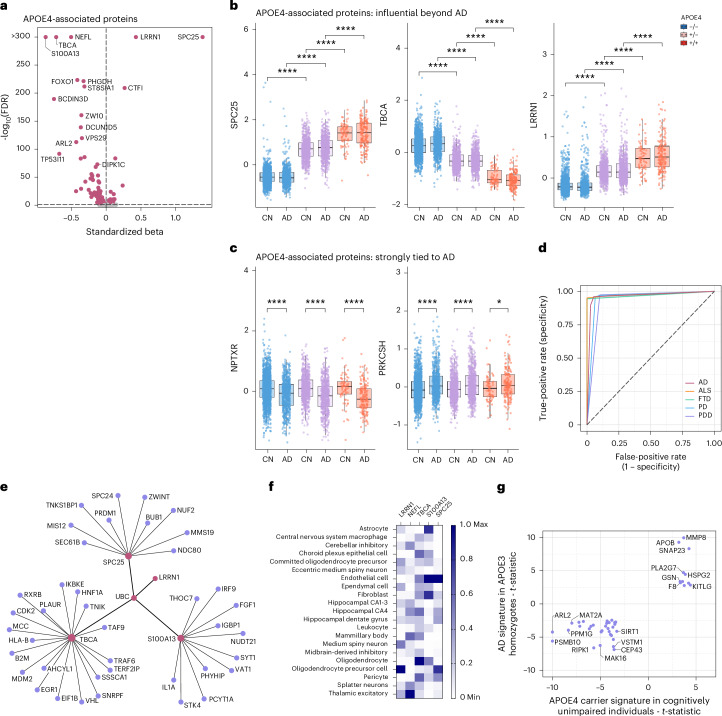
Disease-dependent and disease-independent of *APOE* ε4 on the human proteome. **a**, Volcano plot shows the protein association profile of *APOE* ε4 after adjusting for AD dementia diagnosis, with red representing significant associations (after FDR correction). At the *y* axis, the −log_10_(FDR-adjusted *P* values) > 300 were set to 300 for better visualization. This was done for S100A13, TBCA, NEFL, LRRN1 and SPC25. **b**,**c**, Box plots show plasma protein level changes of the proteins with the strongest *APOE* ε4 associations (**b**) and for *APOE* ε4-associated proteins strongly tied to AD dementia diagnosis (**c**). For **b** and **c**, the *y* axis represents residual protein levels after adjusting for age, sex, mean protein level and contribution site. The center line of each box indicates the median, with lower and upper edges representing the 25th and 75th percentiles. Whiskers extend to the most extreme values within 1.5 times the interquartile range; data points beyond this range were excluded as outliers. The *x* axis represents AD diagnosis. The color indicates *APOE* ε4 carrier status; ‘−/−’ indicates *APOE* ε4 non-carriers; ‘±’ indicates ε3/ε4; and ‘+/+’ indicates ε4/ε4. Welch’s *t*-test was used to compare residual protein levels between groups. Two-sided *P* values are reported. ****P < 0.0001 and *P < 0.05. *P* values were not adjusted for multiple comparisons, as only prespecified group contrasts are shown. Results marked with **** remain significant (pFDR < 0.0001) even after adjustment for multiple comparisons with the Benjamini–Hochberg method, whereas those marked with * do not. **d**, Receiver operating characteristic area under the curve (ROC-AUC) showing the performance of a machine learning model using only five proteins to predict *APOE* ε4 status across different diagnostic groups, in a held-out sample. **e**, Protein interaction network including four of those five proteins (red). **f**, Neural cell type expression of RNA transcripts encoding the five *APOE* ε4-predictive proteins. Plot shows mix-max scaling of protein-coding transcripts per million for each identified APOE ε4 protein. **g**, Correlation of effect sizes for proteins associated with *APOE* ε4 in cognitively unimpaired samples (*x* axis) and AD associated with AD diagnosis in *APOE* ε3/ε3 homozygotes. Limma *t*-statistic is shown for both contrasts; only proteins associated with both *APOE* ε4 and AD (adjusted *P* < 0.05 for both analyses) with the same direction of effect are visualized. For visibility purposes, *t*-statistic values higher than 10 were capped. PDD, Parkinsonʼs disease dementia; pFDR, FDR-corrected *P* value.

Using a combination of machine learning and biological enrichment approaches, we sought to isolate the molecular signatures of *APOE* ε4, the main genetic risk factor for sporadic AD, independent of AD and other conditions. Several proteins, including SPC25, LRRN1, S100A13 and NEFL, were strongly associated with *APOE* ε4 versus other alleles (Fig. 3a), paralleling prior observations for some of these targets in serum^27^. Some proteins, such as SPC25 and LRRN1, showed no difference between AD and Controls but exhibited dose-dependent associations with *APOE* ε4 (Fig. 3b), suggesting that previously identified links to AD from Vignette 1’s differential abundance analysis were driven by *APOE* ε4 enrichment in AD cases. Conversely, well-known AD-associated neuromodulatory proteins such as NPTXR and GDF2 were robustly associated with AD diagnosis, irrespective of *APOE* ε4 allelic dose (Fig. 3c).

Notably, the effects of *APOE* ε4 genotype on the plasma proteome were so robust that a machine learning model with only five proteins (SPC25, NEFL, S100A13, TBCA and LRRN1) predicted *APOE* ε4 carrier status in unseen patients with high accuracy, both within AD and within non-AD Patients (area under the curve (AUC) range, 0.90–0.96; Fig. 3d). Leveraging protein–protein interaction libraries and single brain cell RNA sequencing data from the Human Protein Atlas^28^, we observed that three of these proteins (SPC25, TBCA and S100A13) were central nodes in the protein–protein interaction network (Fig. 3e) and brain cell type expression patterns (Fig. 3f)^28^. Ubiquitin-C, however, was found to be a key connection point across the central node proteins, suggesting potential convergence on proteostatic pathways.

Lastly, to identify potential genotype–phenotype links, we compared proteins associated with *APOE* ε4 in cognitively unimpaired individuals (*n* = 2,817; 215 proteins) to those associated with AD in *APOE* ε3 homozygotes (*n* = 1,843; 2,150 proteins). Forty-four overlapping proteins showed consistent directionality (Fig. 3g), including targets elevated in *APOE* ε4 and AD. Patients involved in immunovascular signaling (MMP8), synaptic vesicle fusion (SNAP23) and lipid trafficking (APOB) pathways relevant to *APOE* biology and cognitive decline. This overlap highlights potential early molecular footprints of AD pathophysiology present even in asymptomatic *APOE* ε4 carriers and underscores the utility of contextualizing by *APOE* genotype when seeking AD-relevant proteomic signals. These shared proteins may reflect core features of *APOE*-related biology that are also prominent in AD, highlighting potential mechanisms through which *APOE* ε4 contributes to disease vulnerability.

## Discussion

The availability of high-dimensional molecular datasets has led to an increasing number of large-scale collaborative programs to share and use these data—a trend forged by the genetics community. Following these data-sharing collaborations, numerous initiatives have led the way in data sharing with the wider scientific community. We would like to highlight in particular two programs focused on neurodegeneration or proteomics. The first large-scale open data-sharing program in neurodegeneration was the Alzheimer’s Disease Neuroimaging Initiative (ADNI): an immensely productive public–private partnership that continues today and has spawned many followers. These include AddNeuroMed/InnoMed, an ADNI-like program in Europe that served as a pilot for the European Union Innovative Medicines Initiative (IMI) funding scheme that itself has generated many data-sharing and sample-sharing programs, including, for example, IMI-EMIF and IMI-EPND in neurodegeneration. In the proteomics arena, the GNPC was preceded by the UK Biobank Pharma Proteomics Project (UKBB-PPP) that generated extensive protein data on 50,000 research participants and is now planning analysis on an additional 250,000 participants, to accompany the extensive clinical, imaging and genomic data available to the scientific community. The GNPC complements both initiatives and many others in providing a disease-focused dataset, as in the ADNI, but at scale, as in the UKBB-PPP.

Notably, a key point of emphasis of the GNPC’s mission is the strong intent to share this dataset with the global research community early in its life cycle. We do not present these analyses as definitive but, rather, as the pilot experiments by a subset of researchers whose datasets contributed to the construction of GNPC V1. We hope this summary paper and more in-depth papers serve as an invitation of collaboration and/or independent analysis from the global community. Only then will we be able to maximize disease insights from GNPC V1 and its combination with other datasets to accelerate translation of insights into the next generation of diagnostics and therapeutics for neurodegenerative diseases.

The initial analyses presented here underscore the versatility and translational potential of the GNPC dataset. First, disease-specific differential abundance and disease-shared clinical severity analyses revealed both established and novel protein targets in plasma across AD, PD and FTD, highlighting shared and distinct biological processes such as vesicle trafficking, synaptic integrity and metabolic dysregulation. These results not only validate previously reported protein markers but also nominate new candidates for mechanistic follow-up and blood biomarker development—an urgent clinical need across diseases. Second, organ aging clocks applied to the GNPC dataset uncovered disease-specific patterns of accelerated aging across brain and peripheral organs, offering a systems-level view of proteomic aging that bridges central and systemic health. These findings extend previous work on biological aging by demonstrating that distinct conditions are associated with unique organ-specific age gaps, supporting their relevance to age-related neurodegenerative diseases. Finally, proteome-wide analysis of the *APOE* genotype revealed a robust and disease-independent *APOE* ε4 signature, with potential mechanistic relevance to proteostasis and lipid transport. These vignettes, each explored further in companion publications^19–21^, illustrate the range of insights made possible by GNPC V1 and set the stage for future hypothesis-driven and exploratory research by the broader scientific community, with the potential to better support trial design, monitoring and subtyping of clinical patients.

These data, as well as these vignette analyses and those in the accompanying papers, suggest that very large protein datasets have potential to add value to drug discovery. Hitherto, the value of genetic data has been increasingly recognized in supporting effective drug discovery. Notably, targets with genetic support are more likely to progress through the drug discovery pipeline^29^ with probability of success recently being calculated to be 2.6 times greater than in targets lacking genetic support, even for genes with small effect sizes^30^. In contrast to the inherited traits represented by genetic variants, proteomics represents biological states. Although genetic factors are intrinsically causal in their relationship with disease, a proteomic association with disease might be consequential of the disease, a factor associated with disease (including response to a therapeutic) or reflect a causal process. Although these observations might suggest a role for proteomics more in supporting drug discovery through biomarker discovery rather than for target identification, the vignettes reported here and in the accompanying papers illustrate the potential for more direct drug target identification/validation. For example, in Vignette 1, the proteomic profiles identified include strong support for synaptic dysfunction with proteins identified that are already clearly part of a mechanism targeted for neurodegeneration drug discovery, such as NPTXR^31^, whereas, in Vignette 3, proteins are identified that are very strongly associated with *APOE* status and with disease state. These proteins will surely now be considered as possible targets for drug discovery, especially considering that the *APOE* genotype is a striking example of a very strong genetic risk factor that has not been the source of equally strong drug discovery programs. The GNPC data also support target validation, an important component of target identification in driving drug discovery. Preclinical models might be used to generate signatures of targets or interventions, and, using GNPC data, these signatures now can be used to predict outcomes. In reverse, signatures or candidate protein targets identified in the GNPC could be validated in such preclinical models. Whether in conjunction with genetic data or with preclinical models, the proteomics data now being made available are likely to become a strong additional component of effective target identification and validation. As additional datasets similar to the GNPC and UKBB-PPP become available, it is possible that proteomics will become as important an element of drug discovery as genetics is today.

Despite the evident success in establishing a substantial dataset, the GNPC has some limitations. These include the relative lack of diversity, reflecting much of past observational bioresources where most research participants have been individuals of European ancestry living in the Global North. The dataset would also be enhanced with other proteomics platforms, including complementary molecular biomarker data including genomics, transcriptomics and metabolomics as well as imaging and clinical data and with other disease types related to neurodegeneration. Data harmonization challenges in a post hoc meta-analysis such as the GNPC include site differences in sample processing, clinical methodologies for diagnoses and patient demographics. For example, incomplete medication information limited the ability to identify and separate definitive drug-induced changes to the proteome from disease-relevant changes and/or to correlate proteomic signatures comprehensively with amyloid and synuclein biomarkers. However, despite the cross-site heterogeneity, we were nonetheless able to identify clear and novel signals in the plasma proteome as presented in the vignettes.

The GNPC has been built to accommodate growth and aims to increase diversity in terms of patients and measurements over time. As a next step, the GNPC is poised to incorporate additional cohorts, samples and data platforms in a V2 dataset and will seek to rectify some of these limitations as we build out the collaboration. The flexibility of the platform allows for secure, iterative data releases to adapt to new discoveries made in the field. The GNPC’s aspiration is to facilitate neurodegenerative disease research and development, driving advances toward better outcomes for people with neurodegenerative diseases through precision, combination therapy optimized for a patient’s disease subtypes.

## Methods

### SomaScan proteomics data processing

Proteomic profiling within the GNPC was primarily performed using the SomaScan platform (SomaLogic). Biofluid specimens were independently shipped by each contributing cohort to SomaLogic, with coordination support provided by Gates Ventures. The SomaScan platform uses slow off-rate modified aptamers (SOMAmers) to quantify thousands of proteins in human biofluids, including plasma, serum and CSF. Samples were analyzed within each contributing cohort using versions 3 (~1,300 targets), 4 (~5,000 targets) or 4.1 (~7,000 targets) of the SomaScan assay.

All participating cohorts confirmed that all contributed clinical and generated biosample data were in compliance with the individual patient consents prior to contributing data to the GNPC. Proteomic data from each contributing cohort were processed separately, following SomaLogic’s standardized adaptive normalization by maximum likelihood (ANML) pipeline for hybridization normalization, signal calibration and quality control. These procedures adjust for systematic variation using internal reference standards and buffer controls included on each assay plate. Between-sample normalization was performed using median signal intensities and adaptive procedures to reduce batch and run-to-run variation. Samples with signal intensities that substantially deviated from expected ranges are flagged by SomaLogic for quality concerns but were not removed from the dataset. After cohort-level processing and normalization, datasets were combined to form the harmonized GNPC V1 dataset.

At the time of release, we have included 53 clinical variables across all datasets and will continue to increase this number toward a target of over 150 harmonizable variables in the next version of the harmonized dataset. The clinical variables included in this first release of data include demographic data, harmonized cognitive data from research assessments and comorbidity data (Supplementary Table 3).

### Clinical and phenotypic data harmonization

To harmonize the clinical metadata across the 23 cohorts, we started with a minimum set of 13 required features that were requested from each cohort: age, gender, years of education, date of visit, diagnosis, date of diagnosis, medication use, comorbidities, vital signs, at least one psychiatric measure, at least one cognitive dementia or functional rating score, at least one cognitive test score and disease-specific genotype data. After initial data contribution, five key vital signs (height, weight, body mass index, resting heart rate and blood pressure) and 14 comorbidities (alcohol use, smoking/tobacco use, stroke, transient ischemic attack, traumatic brain injury, cancer, congestive heart failure, chronic obstructive pulmonary disease, myocardial infarction, atrial fibrillation, angina, hyperlipidemia and hypertension) were identified as common features across at least five cohorts for harmonization. The 19 identified features were then identified, where available, from each of the cohort’s data contributions. Demographic and biometric information was normalized to a common scale. Biologically impossible values were cleaned from the dataset. To handle outlier values in years of education, height, weight, body mass index, resting heart rate, blood pressure and total years smoked, an additional variable was created for each feature to indicate if a value was within 2 or 3 s.d. from the mean. Variables were aligned through a mapping schema, ensuring that equivalent tests and demographic categories were matched correctly.

Diagnosis information and control and cognitive impairment data were included, if provided. Diagnosis data for AD, PD, ALS and FTD were captured from the provided clinical data. Due to the variability in AD diagnosis methodology across the 23 cohorts, the method for diagnosis was provided. Using the provided diagnosis data from each site and CDR test scores, a harmonized Clinical Diagnosis variable was created categorizing each participant into one of four categories: Cognitively Normal (CN) (CDR = 0 or confirmed recruited control participant), MCI (CDR = 0.5), Dementia (CDR ≥ 1 or a confirmed diagnosis of AD or FTD) or Other Neurodegenerative Disease (a confirmed case of PD or ALS). For participants with no formal diagnosis information or CDR reported, cognitive test scores (MMSE or MoCA) were used to categorize an individual’s cognitive impairment as Not Impaired (MMSE ≥ 24 and MoCA ≥ 17) or Impaired (MMSE < 24 and MoCA < 17) (Supplementary Tables 2 and 3). Datasets were merged using unique participant identifiers, followed by quality control checks to rectify inconsistencies. Finally, validation was performed by comparing results from the integrated dataset with original study findings.

### Vignette approach

Each of the three vignettes presented here was conducted by separate workstreams within the GNPC, each using distinct criteria and methodological frameworks tailored to their specific research questions. These analyses were intentionally designed to highlight the breadth of analytic approaches enabled by the GNPC dataset—from disease-specific differential abundance and transdiagnostic clinical severity modeling to biological aging clocks and genotype-based signatures. As such, they serve as illustrative examples rather than a unified analytic pipeline, showcasing the flexibility and depth of the GNPC V1 resource for diverse scientific inquiries.

### Vignette 1 methods: disease-specific differential abundance

#### Sample selection and inclusion criteria

For AD, PD, FTD and ALS, we used protein data from each participant’s first available plasma sample, based on clinical data from the cohorts. To reduce diagnostic ambiguity, we excluded participants with conflicting or overlapping clinical labels. This included individuals labeled with both AD and mild cognitive impairment (MCI)/subjective cognitive impairment (SCI) (*n* = 271) and those assigned two distinct neurodegenerative disease diagnoses (*n* = 90). We opted to include only individuals diagnosed with AD at the dementia stage, thus excluding those in prodromal stages. CN Control participants were included if they were explicitly labeled as Controls in their respective cohorts and/or had a CDR score of 0. We also removed participants missing age or sex data (*n* = 54). After all exclusions, the final dataset comprised 5,879 Controls, 1,966 patients with AD, 607 patients with PD, 175 patients with FTD and 254 patients with ALS (Supplementary Table 4).

#### Protein data processing

Protein aptamer abundance levels (relative fluorescence units) were log_2_ transformed prior to analysis. Extreme outliers, defined as values more than 5 s.d. above or below the mean across the full dataset, were removed for each aptamer. To ensure biological relevance, only aptamers targeting human proteins were retained for analysis, resulting in a final analytic set of 7,289 unique aptamers (Supplementary Table 5).

#### Cohort-specific differential abundance and meta-analysis

Given the inherent heterogeneity across contributing GNPC cohorts, all differential abundance analyses were first conducted within each cohort. For each neurodegenerative disease, Patient samples were compared to Control samples using linear regression with the protein used as the dependent variable and disease diagnosis as the independent variable, with age and sex included as covariates. Only cohorts with at least five patients in each diagnostic category (AD, PD, FTD or ALS) were analyzed. In the few cohorts that lacked internal control samples (for example, Cohorts R and G for AD, Cohorts R and T for PD and Cohort S for ALS), patient data were compared to pooled Controls from the remaining cohorts. See Supplementary Table 3 for a breakdown of Controls and Patients across cohorts.

After estimating effect sizes within each cohort, we conducted fixed-effects meta-analyses to identify proteins with reproducible disease associations across cohorts, thereby providing internal replication of top signals. For each aptamer, a weighted average of effect sizes was calculated, taking into account the sample size of patients at each cohort, and meta-analytic *P*values were computed using the weighted *z-*score method via the ‘metapro R’ package. These *P*values were then adjusted for multiple comparisons using both FDR and Bonferroni correction (separately). To further assess reproducibility, we calculated the number of cohorts in which each aptamer was significantly differentially abundant (FDR < 0.05) as well as the consistency of directionality (that is, the number of cohorts showing concordant upregulation or downregulation for a given target.)

#### Pathway enrichment analysis

Proteins identified in the meta-analytic differential abundance analysis were filtered into gene set enrichment analysis using the R libraries ‘ReactomePA’ and ‘clusterProfiler’. Proteins were selected based on Bonferroni cutoffs where possible (AD and PD comparisons). For FTD and ALS, where statistical power was more limited, we applied a less stringent threshold of FDR < 0.05. Enrichment analysis was performed with the Reactome library against a background universe consisting of 6,404 unique human proteins measured on the SomaScan 7K platform.

#### Development of transdiagnostic signature of cognitive decline

Independent of diagnostic category, we also sought to identify a plasma proteomic signature of clinical severity across the GNPC disease continuum by leveraging the subset of Patients with global CDR scales (*N* = 6,187) and cognitive test scores (*N* = 5,969). To model the full spectrum of clinical severity, inclusion criteria were intentionally broadened to include all AD, PD and FTD Patients ranging from CDR = 0.5 to CDR = 3. CDR global scores were modeled based on a five-level ordinal stage from no impairment (CDR = 0) to severe impairment (CDR = 3). A proteome-wide association analysis for CDR global scores was conducted by age (linear and quadratic), sex, their interactions and smoking/alcohol status. Significant proteins (FDR < 0.05) were used to construct a multivariate protein signature via LASSO regression, with data split into 70% training and 30% test sets. Diagnosis-stratified models also tested the multivariate protein signature’s ability to track cognitive severity across each clinical condition. To demonstrate robustness of prediction to orthogonal clinical measures, the CDR-derived protein signature was modeled as a predictor of cognitive performance using cross-walked, harmonized MoCA/MMSE scores (0–30 scale).

### Vignette 2 methods: organ aging across diseases

A series of recent studies has generated compelling evidence for the use of molecular ‘clocks’ to estimate biological aging at the organismal and organ-specific level using plasma proteomics. Building upon this foundation, we applied validated organ age models to the GNPC dataset to evaluate disease-specific patterns of accelerated organ aging^32^.

#### Sample selection and inclusion criteria

This analysis was restricted to nine GNPC cohorts that included both Patients and CN Control participants, as control data were required to compute normative organ aging estimates. Diagnostic groups included individuals with AD (*n* = 1,973), FTD (*n* = 151) and PD (*n* = 334).

#### Protein data processing

Protein levels from the SomaScan version 4.1 platform were normalized to match the version 4.0 reference using internal reference-based scaling methods, enabling consistent application of the organ aging models across assays.

#### Organ age estimation and disease associations

Organ age estimates were computed for brain, liver, kidney, muscle, adipose, immune, lung, intestine, artery, pancreas and heart tissues, using established organ aging models that use proteins specific to each organ. Cognition-enriched brain age estimates were derived using the CognitionBrain model, which additionally limits the brain-specific proteins used for age estimation to only those that are important for the association of the model age gap with cognitive impairment, as determined by the Feature Importance for Biological Aging (FIBA) algorithm. Additionally, whole-organism (‘organismal’) age estimates, using only proteins common across organs, and ‘conventional’ age estimates, using all measured proteins regardless of tissue enrichment, were also calculated. The organ age gap was derived as the difference between predicted age and the cohort-specific locally weighted scatterplot smoothing (LOWESS) regression estimate for individuals with a normal cognitive clinical diagnosis (Supplementary Table 9). Associations between *z*-score normalized organ age gaps and diagnosis (AD, PD and FTD) were determined using logistic regression models, adjusting for age, sex and cohort.

### Vignette 3 methods: *APOE* proteome

The *APOE* ε4 allele is the leading genetic risk factor of late-onset AD, yet its high frequency in patients with AD complicates efforts to disentangle *APOE* from AD-related proteomic changes. To address this, we implemented a stepwise analytic framework to isolate proteomic signatures linked to *APOE* ε4 carriership, irrespective of AD and other neurodegenerative conditions.

#### Sample selection and inclusion criteria

Participants were selected from GNPC cohorts based on strict diagnostic and cognitive performance criteria to ensure well-defined AD and Control groups. We first identified individuals labeled with an AD dementia diagnosis or recruited as Controls. Participants with multiple neurodegenerative disease diagnoses were excluded. Patients with AD dementia were required to have either MMSE < 24 or MoCA <17 and CDR ≥ 1, to ensure alignment with established diagnostic thresholds for dementia. CN participants did not meet criteria for any clinical diagnosis (for example, AD, MCI, FTD, ALS and PD) and had CDR = 0 and, at a minimum, cognitive test scores above a dementia threshold (MMSE ≥ 24 and/or MoCA ≥ 17) (Supplementary Table 10). To minimize confounding from opposing genotype effects, *APOE* ε2/ε4 heterozygotes were excluded from the analysis.

After applying the above criteria, a total of 3,934 participants were identified for further analysis. Among them, *n* = 2,357 were *APOE* ε4 non-carriers (ε4^−^) and *n* = 1,577 were *APOE* ε4 carriers (ε4^+^, either ε3/ε4 or ε4/ε4). At baseline, 1,438 individuals were diagnosed with AD, and the remaining 2,496 were non-impaired (CN) Controls.

#### *APOE* ε4 association analysis

Consistent with differential abundance analyses (see Vignette 1 methods), log_2_-transformed aptamer values that deviated more than 5 s.d. from the mean were defined as outliers and excluded. Proteins associated with *APOE* ε4 carriers (carrier versus non-carrier) were identified using linear regression models, adjusting for AD dementia diagnosis (CN versus AD dementia diagnosis), age, sex, contributing cohort code and mean overall protein level (Supplementary Table 11). Multiple testing correction was performed using FDR adjustment (Benjamini–Hochberg method, *α* = 0.05). Residualized protein values were used for visualization. The Wilcoxon rank-sum test in the ‘ggpubr’ R package (version 0.6.0) was used to test and mark the changes in protein residuals in the *APOE* ε4 carrier group and the AD dementia diagnosis group (Supplementary Table 12)^33^.

#### Machine learning classification of *APOE* ε4 status

To identify a disease-agnostic proteomic signature of *APOE ε*4 allele status, a machine learning classification model was developed. Proteomic data were split into training (70%) and test (30%) sets, with standardization performed independently within each set. A classification and regression trees (CART) approach was applied to the data using cross-validation and fine tuning. All final metrics reported were derived from application of the model to the left-out testing dataset.

Model performance was stratified by CN and AD as well as FTD, PD and Parkinsonʼs disease dementia. First-order functional networks were built from proteins of interest identified by the CART model. In addition, brain cell subtype-specific enrichment analysis was performed using single-nuclei brain RNA sequencing data from the Human Protein Atlas.

#### Overlap of *APOE*-associated and AD-associated proteins

To explore proteins influenced independently by *APOE ε*4 carriership and AD diagnosis, two additional comparisons were performed. First, proteins associated with *APOE* ε4 in cognitively unimpaired individuals were identified. Second, proteins associated with AD compared to Controls were identified specifically in *APOE* ε3 homozygote AD patients. Analyses were performed using limma (limma_3.62.2) and adjusted for sex, age at visit and contributor code. Multiple testing correction was performed using FDR adjustment (Benjamini–Hochberg method, *α* = 0.05).

### Reporting summary

Further information on research design is available in the Nature Portfolio Reporting Summary linked to this article.

## Online content

Any methods, additional references, Nature Portfolio reporting summaries, source data, extended data, supplementary information, acknowledgements, peer review information; details of author contributions and competing interests; and statements of data and code availability are available at 10.1038/s41591-025-03834-0.

## Supplementary information

Supplementary Information Supplementary Fig. 1 and list of GNPC members.

Reporting Summary

Supplementary Tables Supplementary Tables 1–12.

## Data Availability

The harmonized GNPC data used to generate these findings were provided to consortium members in June 2024 and will be made available for public request by the AD Data Initiative in July 2025. Members of the global research community will be able to access the metadata and place a data use request via the AD Discovery Portal (https://discover.alzheimersdata.org/). Access is contingent upon adherence to the GNPC Data Use Agreement and the Publication Policies.

## Code availability

All custom code and data that were employed for this paper are saved within the GNPC AD Workbench. All custom code central to the findings in this paper will be made available outside of private workspaces upon publication.
